# Imperial strategy of cancer cells through mitochondrial transfer

**DOI:** 10.1002/1878-0261.70142

**Published:** 2025-10-05

**Authors:** Takamasa Ishino, Yosuke Togashi

**Affiliations:** ^1^ Faculty of Medicine, Department of Tumor Microenvironment Dentistry and Pharmaceutical Sciences, Okayama University Japan; ^2^ Department of Respiratory Medicine Okayama University Hospital Japan; ^3^ Faculty of Medicine, Kindai University Osaka Japan

**Keywords:** cancer‐associated fibroblast, mitochondrial transfer, tumor microenvironment

## Abstract

Mitochondria are essential organelles that regulate various biological processes including metabolism. Beyond their intracellular functions, intercellular mitochondrial transfer has emerged as a novel mechanism of intercellular communication. Notably, an increasing number of studies have reported its occurrence in the tumor microenvironment (TME), where it contributes to tumor progression. While previous studies largely characterized cancer cells as recipients of mitochondria, Cangkrama et al. demonstrated that cancer cells donate their mitochondria to fibroblasts via tunneling nanotubes. The mitochondrial transfer to fibroblasts reprogrammed them into cancer‐associated fibroblasts exhibiting combined myofibroblastic and inflammatory characteristics, with enhanced oxidative metabolism and pro‐tumorigenic activity. Our group has identified mitochondrial ‘hijack’ from cancer cells to tumor‐infiltrating lymphocytes, leading to an impaired antitumor immunity. These insights underscore the need to recognize cancer cells as mitochondrial donors in the TME capable of reshaping the TME to their own advantage, resembling a dynastic expansion strategy that exerts influence by strategically placing lineages.

## Graphical abstract

Cangkrama et al. demonstrated that cancer cells donate their mitochondria to fibroblasts through mitochondrial transfer, reprogramming them into ‘MitoCAF’. Likewise, our group has identified mitochondrial transfer from cancer cells to tumor infiltrating lymphocytes, resulting in mitochondrial ‘hijack’ and impaired antitumor immunity. These findings highlight the need to consider cancer cells as mitochondrial donors within the tumor microenvironment.

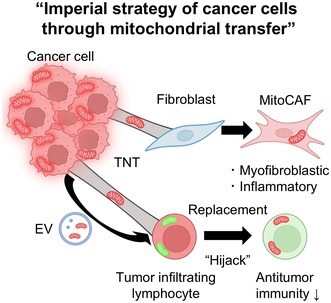

**Abbreviations**
CAF: cancer‐associated fibroblast
EV: extracellular vesicle
mtDNA: mitochondrial DNA
ROS: reactive oxygen species
TIL: tumor infiltrating lymphocytes
TME: tumor microenvironment
TNT: tunneling nanotube

Mitochondria are organelles of eukaryotic cells, which play key roles in cellular metabolism as well as biosynthesis, redox balance, calcium homeostasis, reactive oxygen species (ROS) control, signal transduction, and the regulation of cell death [1]. Based on the endosymbiotic theory, mitochondria are considered to derive from prokaryotes that were engulfed by primitive eukaryotic cells and subsequently evolved into organelles [1]. Since intercellular mitochondria transfer was first reported in 2006 [2], mitochondrial transfer within the tumor microenvironment (TME) has been increasingly reported. Though the mechanisms of mitochondrial transfer are not fully understood, they are generally classified into contact‐dependent and contact‐independent mechanisms based on current evidence. Contact‐dependent transfer reportedly occurs via tunneling nanotubes (TNTs), dendritic projections, or adhesion, whereas contact‐independent transfer is reported to involve mitochondria released within extracellular vesicles (EVs) or as free mitochondria [3]. Mitochondria have been reported to transfer to cancer cells from a variety of donor cells, including cancer cells [4], immune cells [5], stromal cells such as cancer‐associated fibroblasts (CAFs), platelets, and neurons [6, 7, 8, 9, 10, 11], which can contribute to cancer pathogenesis. In contrast, we have recently reported mitochondrial transfer from cancer cells to tumor infiltrating lymphocytes (TILs) through TNTs and EVs as a mechanism to suppress antitumor immune responses [12]. In addition, Cangkrama et al. recently reported that mitochondrial transfer from cancer cells to fibroblasts fosters their differentiation into CAFs exhibiting combined myofibroblastic and inflammatory features [13].

Given the association of the CAF phenotype with metabolic alterations [14], Cangkrama, et al. cocultured cancer cells with fibroblasts and demonstrated mitochondrial transfer from cancer cells mediated via TNTs. The mitochondrial transfer was also confirmed in xenograft tumors, where human cancer cell‐derived mitochondria were found in murine fibroblasts, highlighting *in vivo* relevance. Fibroblasts that received cancer cell mitochondria showed metabolic reprogramming with increased oxidative phosphorylation, ATP and ROS production. The mitochondrial transfer from cancer cells reprogrammed fibroblasts into a hybrid CAF phenotype (termed ‘MitoCAF’), characterized by induction of myofibroblastic (*ACTA2*, *COL1A1*, and *INHBA*) and inflammatory (*IL‐6*) markers, whereas antigen presenting markers (MHC class II, CD74) were not upregulated. The reprogrammed fibroblasts secreted pro‐tumorigenic cytokines and extracellular matrix components that promoted cancer cell growth, migration, and angiogenesis. The reprogramming of fibroblasts depended on the integrity of the transferred mitochondria because transfer of dysfunctional mitochondria did not trigger CAF differentiation. Mechanistically, this study identified the mitochondrial trafficking GTPase MIRO2 as a key regulator of the mitochondrial transfer from cancer cells to fibroblasts. MIRO2 was required in donor cancer cells for TNT‐mediated mitochondrial transfer. Clinically, MIRO2 expression was enriched at the invasive front of human carcinomas.

## 1 Significance and future perspectives

Cangkrama et al. suggested that MIRO2‐mediated mitochondrial transfer from cancer cells can drive fibroblast reprogramming into CAFs with myofibroblastic and inflammatory characteristics, highlighting mitochondrial communication as a novel aspect of tumor–stroma interactions [13]. In addition, we have recently reported not only mitochondrial transfer but also replacement from cancer cells to TILs (‘Hijack’) [12]. In particular, the replacement by cancer cell‐derived functionally impaired mitochondria with mitochondrial DNA (mtDNA) mutations resulted in cellular senescence and/or terminal exhaustion in TILs, leading to impaired antitumor immunity [12]. These phenomena resemble a dynastic expansion strategy, whereby power was consolidated across countries through the placement of lineage into different royal houses. Taken together, we consider it is necessary to think of cancer cells not only as mitochondrial recipients but also as donors in the TME. In this context, it is important to consider both the mitochondrial function of donor cancer cells and the recipient cell types.

As a method to identify mitochondrial transfer in the article [13], several approaches have been employed, such as mitochondrial dyes, mitochondria reporter systems, and mtDNA analysis. Given the strengths and weaknesses of each method, combining complementary approaches is necessary for reliable assessment [3, 15]. Although mitochondrial dyes such as MitoTracker have been widely used, recent reports have highlighted notable concerns of false positives, underscoring the need for careful interpretation [3, 15, 16]. Thus, we consider Cangkrama et al. experimentally demonstrated mitochondrial transfer to fibroblast with accuracy. Nonetheless, the *in vivo* validation was performed in an immunodeficient model with human cancer cells and mouse fibroblasts, which did not accurately represent the TME and lacked syngeneic evaluation. Indeed, the complementary effect of mitochondria from different species is reportedly transient and not sustained in the long term [17]. It has also been reported that differences in mtDNA alone can activate the innate immune system [18]. In addition, the study focused only on mitochondrial transfer to fibroblasts and did not assess transfer to other stromal or immune cells. These observations underscore the need to examine mitochondrial transfer in syngeneic models. Furthermore, direct evidence of mitochondrial transfer from cancer cells to fibroblasts using clinical samples also remains an important challenge for future research. Recent technological advances have enabled single‐cell‐level analysis of mtDNA, which is expected to drive significant progress in the mitochondrial research field [19, 20]. We consider it is essential to apply such novel technologies to clinical samples in order to deepen our understanding of intercellular mitochondrial transfer. In summary, intercellular mitochondrial communication through mitochondrial transfer is expected to provide new insights into the pathogenesis of various diseases including cancer, leading to the development of novel biomarkers and therapeutic strategies.

## Conflict of interest

YT received institutional research funding and honoraria from AstraZeneca, and Chugai Pharmaceutical; institutional research funding from Daiichi‐Sankyo, Janssen Pharmaceutical, KORTUC, Takeda Pharmaceutical and Taiho Pharmaceutical; and honoraria from Ono Pharmaceutical, Bristol‐Myers Squibb, Eisai and MSD outside of this study.

## Author contributions

TI and YT co‐wrote this commentary.

## References

[1] Spinelli JB , Haigis MC . The multifaceted contributions of mitochondria to cellular metabolism. Nat Cell Biol. 2018;20:745–754. 29950572 10.1038/s41556-018-0124-1 PMC6541229

[2] Spees JL , Olson SD , Whitney MJ , Prockop DJ . Mitochondrial transfer between cells can rescue aerobic respiration. Pro Natl Aca Sci USA. 2006;103:1283–1288. 10.1073/pnas.0510511103 PMC1345715 16432190

[3] Brestoff JR , Singh KK , Aquilano K , Becker LB , Berridge MV , Boilard E , et al. Recommendations for mitochondria transfer and transplantation nomenclature and characterization. Nat Metab. 2025;7:53–67. 39820558 10.1038/s42255-024-01200-x

[4] Takenaga K , Koshikawa N , Nagase H . Intercellular transfer of mitochondrial DNA carrying metastasis‐enhancing pathogenic mutations from high‐ to low‐metastatic tumor cells and stromal cells via extracellular vesicles. BMC Mol Cell Biol. 2021;22:52. 34615464 10.1186/s12860-021-00391-5 PMC8496074

[5] Saha T , Dash C , Jayabalan R , Khiste S , Kulkarni A , Kurmi K , et al. Intercellular nanotubes mediate mitochondrial trafficking between cancer and immune cells. Nat Nanotechnol. 2022;17:98–106. 34795441 10.1038/s41565-021-01000-4 PMC10071558

[6] Pasquier J , Guerrouahen BS , Al Thawadi H , Ghiabi P , Maleki M , Abu‐Kaoud N , et al. Preferential transfer of mitochondria from endothelial to cancer cells through tunneling nanotubes modulates chemoresistance. J Transl Med. 2013;11:94. 23574623 10.1186/1479-5876-11-94 PMC3668949

[7] Burt R , Dey A , Aref S , Aguiar M , Akarca A , Bailey K , et al. Activated stromal cells transfer mitochondria to rescue acute lymphoblastic leukemia cells from oxidative stress. Blood. 2019;134:1415–1429. 31501154 10.1182/blood.2019001398 PMC6856969

[8] Ippolito L , Morandi A , Taddei ML , Parri M , Comito G , Iscaro A , et al. Cancer‐associated fibroblasts promote prostate cancer malignancy via metabolic rewiring and mitochondrial transfer. Oncogene. 2019;38:5339–5355. 30936458 10.1038/s41388-019-0805-7

[9] Salaud C , Alvarez‐Arenas A , Geraldo F , Belmonte‐Beitia J , Calvo GF , Gratas C , et al. Mitochondria transfer from tumor‐activated stromal cells (TASC) to primary Glioblastoma cells. Biochem Biophys Res Commun. 2020;533:139–147. 32943183 10.1016/j.bbrc.2020.08.101

[10] Watson DC , Bayik D , Storevik S , Moreino SS , Sprowls SA , Han J , et al. GAP43‐dependent mitochondria transfer from astrocytes enhances glioblastoma tumorigenicity. Nat Cancer. 2023;4:648–664. 37169842 10.1038/s43018-023-00556-5 PMC10212766

[11] Hoover G , Gilbert S , Curley O , Obellianne C , Lin MT , Hixson W , et al. Nerve‐to‐cancer transfer of mitochondria during cancer metastasis. Nature. 2025;644:252–262. 40562940 10.1038/s41586-025-09176-8 PMC12328229

[12] Ikeda H , Kawase K , Nishi T , Watanabe T , Takenaga K , Inozume T , et al. Immune evasion through mitochondrial transfer in the tumour microenvironment. Nature. 2025;638:225–236. 39843734 10.1038/s41586-024-08439-0 PMC11798832

[13] Cangkrama M , Liu H , Wu X , Yates J , Whipman J , Gäbelein CG , et al. MIRO2‐mediated mitochondrial transfer from cancer cells induces cancer‐associated fibroblast differentiation. Nat Cancer. 2025;1–20. 40877413 10.1038/s43018-025-01038-6 PMC12559006

[14] Sahai E , Astsaturov I , Cukierman E , DeNardo DG , Egeblad M , Evans RM , et al. A framework for advancing our understanding of cancer‐associated fibroblasts. Nat Rev Cancer. 2020;20:174–186. 31980749 10.1038/s41568-019-0238-1 PMC7046529

[15] Tiash S , Brestoff JR , Crewe C . A guide to studying mitochondria transfer. Nat Cell Biol. 2023;25:1551–1553. 37853133 10.1038/s41556-023-01246-1 PMC11610514

[16] Neikirk K , Marshall AG , Kula B , Smith N , LeBlanc S , Hinton A . MitoTracker: A useful tool in need of better alternatives. Eur J Cell Biol. 2023;102:151371. 37956476 10.1016/j.ejcb.2023.151371

[17] Yoon YG , Haug CL , Koob MD . Interspecies mitochondrial fusion between mouse and human mitochondria is rapid and efficient. Mitochondrion. 2007;7:223–229. 17251069 10.1016/j.mito.2006.11.022 PMC2693707

[18] Ishikawa K , Toyama‐Sorimachi N , Nakada K , Morimoto M , Imanishi H , Yoshizaki M , et al. The innate immune system in host mice targets cells with allogenic mitochondrial DNA. J Exp Med. 2010;207:2297–2305. 20937705 10.1084/jem.20092296 PMC2964578

[19] Nitsch L , Lareau CA , Ludwig LS . Mitochondrial genetics through the lens of single‐cell multi‐omics. Nat Genet. 2024;56:1355–1365. 38951641 10.1038/s41588-024-01794-8 PMC11260401

[20] Zhang H , Yu X , Ye J , Li H , Hu J , Tan Y , et al. Systematic investigation of mitochondrial transfer between cancer cells and T cells at single‐cell resolution. Cancer Cell. 2023;41:1788–1802. 37816332 10.1016/j.ccell.2023.09.003 PMC10568073

